# Anwendung der extra- und intracochleären Elektrocochleographie während und nach der Cochleaimplantation

**DOI:** 10.1007/s00106-024-01481-4

**Published:** 2024-05-18

**Authors:** Adrian Dalbert, Stefan Weder

**Affiliations:** 1 Klinik für Ohren‑, Nasen‑, Hals- und Gesichtschirurgie, Universitätsspital Zürich, Zürich, Schweiz; 2 Universitätsklinik für Hals‑, Nasen- und Ohrenkrankheiten, Kopf- und Halschirurgie Inselspital, Universitätsspital Bern, Freiburgstrasse 20, 3012 Bern, Schweiz

**Keywords:** Elektrocochleographie, Cochleaimplantat, Schwerhörigkeit, Restgehör, Cochleäre Funktion, Electrocochleography, Cochlear implant, Hearing loss, Residual hearing, Cochlear function

## Abstract

Die Elektrocochleographie (ECochG) bietet eine aussichtsreiche Möglichkeit zur Überwachung der cochleären Funktion während der Cochleaimplantation und zur Erforschung der Ursachen des Verlusts cochleärer Restfunktion nach der Implantation. Die vorliegende Arbeit gibt einen Überblick über den aktuellen Forschungs- und Anwendungsstand der ECochG, sowohl während als auch nach der Cochleaimplantation. Die intraoperative ECochG kann entweder durch das Implantat selbst oder mittels einer extracochleären Messelektrode durchgeführt werden. Postoperative ECochG-Aufnahmen sind über das Implantat möglich. Verschiedene Studien haben gezeigt, dass ein signifikanter Abfall der ECochG-Amplitude während der Elektrodeninsertion mit einem erhöhten Risiko für den Verlust der cochleären Restfunktion korreliert, wobei bedeutsame cochleäre Ereignisse vornehmlich gegen Ende der Insertion auftreten. Postoperative Daten deuten darauf hin, dass der Verlust der cochleären Funktion hauptsächlich in der frühen postoperativen Phase erfolgt. Zukünftige Forschungsansätze umfassen die Automatisierung und Objektivierung der Signalauswertung sowie eine vertiefte Untersuchung der den Signaländerungen zugrunde liegenden Mechanismen.

## Abstract (en)

Electrocochleography (ECochG) represents a promising approach for monitoring cochlear function during cochlear implantation and for investigating the causes of residual cochlear function loss after implantation. This paper provides an overview of the current research and application status of ECochG, both during and after cochlear implantation. Intraoperative ECochG can be conducted either via the implant itself or an extracochlear measuring electrode. Postoperative ECochG recordings are also feasible via the implant. Various studies have demonstrated that a significant decrease in ECochG amplitude during electrode insertion correlates with an increased risk of losing residual cochlear function, with critical cochlear events occurring primarily towards the end of the insertion. Postoperative data suggest that the loss of cochlear function mainly occurs in the early postoperative phase. Future research directions include the automation and objectification of signal analysis, as well as a more in-depth investigation into the underlying mechanisms of these signal changes.

Eine möglichst schonende Einlage des Cochleaimplantats (CI) wird heutzutage grundsätzlich bei allen Operationen angestrebt. Die Elektrocochleographie (ECochG) stellt in diesem Zusammenhang ein vielversprechendes Instrument dar, um die Innenohrfunktion vor, während oder nach der Implantation zu überwachen. Der Einsatz von ECochG bei CI-Empfängern ermöglicht ein umfassenderes Verständnis der Innenohrfunktion und unterstützt die Identifikation von Faktoren, die zu einem Funktionsverlust des Innenohrs führen können. Der vorliegende Artikel liefert eine Zusammenfassung des gegenwärtigen Forschungsstands sowie der praktischen Anwendungen der ECochG.

## Überwachungsinstrument für die cochleäre Funktion 

Die Elektrocochleographie (ECochG) ist ein diagnostisches Verfahren zur Erfassung von Innenohrpotenzialen als Reaktion auf akustische Stimuli. Es wird zunehmend bei Kandidaten für Cochleaimplantate (CI) und bei CI-Trägern eingesetzt, um die residuale Innenohrfunktion während und nach der Implantation zu beurteilen. Die Bedeutung von ECochG-Aufzeichnungen wächst, insbesondere da immer mehr CI-Kandidaten über Resthörvermögen verfügen. Es hat sich gezeigt, dass Patienten, bei denen das Resthörvermögen postoperativ erhalten bleibt, ein verbessertes Sprachverständnis, ein natürlicheres Hörerlebnis und eine bessere räumliche Hörfähigkeit aufweisen im Vergleich zu denen, die ausschließlich auf die elektrische Hörwahrnehmung angewiesen sind [1]. Intraoperative ECochG-Messungen bieten wertvolle Hinweise auf potenzielle Störungen oder Schädigungen der cochleären Funktionen während der Elektrodenplatzierung [2]. Angesichts dieser Erkenntnisse haben diverse Forschungszentren es sich zum Ziel gesetzt, die ECochG als Überwachungsinstrument für die cochleäre Funktion in der klinischen Routine zu implementieren und so die Wahrscheinlichkeit des Erhalts des akustischen Hörens zu erhöhen.

ECochG-Messungen können über das Implantat oder eine separate Messelektrode erfolgen

Die Durchführung von ECochG-Messungen kann entweder über das Implantat selbst (intracochleäre ECochG, [3]) oder über eine separate Messelektrode, die auf das Promontorium aufgesetzt wird (extracochleäre ECochG, [4]), erfolgen. Die Messung ist während der Einführung der Elektrode, nach der vollständigen Insertion oder zu einem beliebigen postoperativen Zeitpunkt im Wachzustand möglich. ECochG beinhaltet 4 primäre Signalkomponenten mit unterschiedlichen Signal-Rausch-Verhältnissen, was die jeweilige Identifikation beeinflusst und somit eine sorgfältige Analyse und Interpretation erforderlich macht.

Eine sachgerechte Anwendung von ECochG-Messungen im klinischen Alltag verlangt ein fundiertes Verständnis der verschiedenen Signalkomponenten, des Messaufbaus sowie der Vor- und Nachteile der intra- und extracochleären Messung. Ebenso ist eine Kenntnis der angewandten Analysemethoden unerlässlich. Die vorliegende Arbeit erörtert diese Aspekte und strebt an, das Verständnis für ECochG-Ableitungen zu vertiefen.

## Erklärung der einzelnen Signalkomponenten

**Abb. 1:**
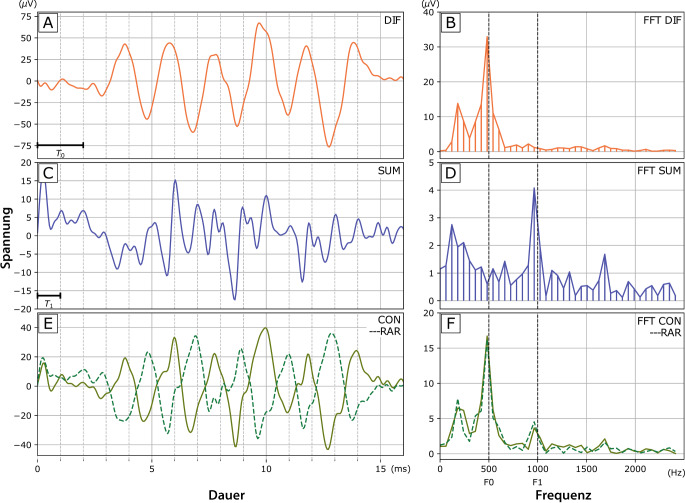
Elektrocochleographie-Messung. Erläuterung s. Text. *x‑Achse* Zeit (ms), *y‑Achse* Spannungsunterschied (µV). Elektrophysiologische Antwort des Innenohrs auf einen 500-Hz-Stimulus, in 2 Polaritäten präsentiert: in positiver (*E*, *durchgezogene Linie*) und in negativer Ausrichtung (*E*, *gestrichelte Linie*). Addition dieser beiden Kurven: Summenpotenzial (*C*), „auditory nerve neurophonic“. Subtraktion der beiden Kurven: Differenzantwort (*A*), „cochlear microphonic“. Abbildungen *B*, *D* und *F* Fourier-Transformationen dieser Antworten (Mit freundl. Genehmigung © Raphael Andonie, alle Rechte vorbehalten)

Bei der Durchführung der ECochG kommen vorrangig Reintöne als Stimuli zum Einsatz, obgleich auch die Verwendung breitbandiger Stimuli möglich ist [5, 6]. Diese Stimuli werden in 2 unterschiedlichen Polaritäten, „rarefaction“ und „condensation“, präsentiert, woraufhin die Reaktionen des Innenohrs erfasst und analysiert werden. Durch Subtraktion oder Addition der Antworten auf diese Polaritäten lassen sich verschiedene Komponenten der gemessenen Antworten differenzieren, wobei zu beachten ist, dass es sich um eine Mischantwort handelt (Abb. 1). Auf der x‑Achse ist die Zeit in Millisekunden (ms) abgetragen, während die y‑Achse den Spannungsunterschied in Mikrovolt (µV) anzeigt. Diese Darstellung zeigt die elektrophysiologische Antwort des Innenohrs auf einen 500 Hz Stimulus, der in zwei Polaritäten präsentiert wird: einmal in positiver (E, blaue Kurve) und einmal in negativer Ausrichtung (E, graue Kurve). Die Addition dieser beiden Kurven resultiert im Summenpotenzial (C), auch bekannt als „auditory nerve neurophonic“. Die Subtraktion der beiden Kurven führt zur Differenzantwort (A), die oft als „cochlear microphonic“ bezeichnet wird. Die Abbildungen B, D und F zeigen jeweils die Fourier-Transformationen dieser Antworten, welche die spezifischen Frequenzanteile darstellen.

Die ECochG umfasst 4 verschiedene Signalkomponenten [7, 8]. Die „cochlear microphonic“ (CM) ist aufgrund ihrer hohen Amplitude und des guten Signal-Rausch-Abstands die am häufigsten untersuchte Komponente. Sie wird primär den äußeren Haarzellen zugeordnet und spiegelt bei Reintönen die Frequenz des Stimulus wider, d. h., bei einem Stimulus von 500 Hz findet sich die CM-Antwort ebenfalls in diesem Frequenzbereich.

Durch die Addition der 2 Polaritätsantworten lässt sich die „auditory nerve neurophonic“ (ANN) isolieren, eine frühe neurale Antwort, die im Frequenzbereich der zweiten Harmonischen des Stimulus liegt. So erwartet man bei einem 500-Hz-Stimulus eine ANN-Antwort um 1000 Hz [9]. Die Amplitude und der Signal-Rausch-Abstand der ANN sind i. Allg. niedriger als die der CM, und die beiden Signale können sich überlagern.

Das „compound action potential“ (CAP) ist eine weitere neurale Antwort und repräsentiert die summierten elektrischen Aktionspotenziale zahlreicher Nervenfasern des Hörnervs, die als Auslenkung kurz nach dem Reiz sichtbar werden. Das Vorhandensein des CAP bei ECochG-Reintonstimulationen ist variabel und hängt sowohl vom Stimulus als auch vom Restgehör ab, insbesondere in den höheren Frequenzen [10].

Das „summating potential“ (SP) schließlich wird als eine Gleichstromantwort verstanden, die hauptsächlich der nichtlinearen Bewegung der Basilarmembran bei höheren Stimulusintensitäten entspricht [11].

Die Analyse mehrerer Komponenten kann die Untersuchung der cochleären Restfunktion verbessern

Insgesamt kann gesagt werden, dass jede dieser 4 Signalkomponenten auf unterschiedliche Weise Einblick in die Funktion des Innenohrs bietet. Die Analyse mehrerer Komponenten kann zur verbesserten Untersuchung der cochleären Restfunktion beitragen [9].

## Messaufbau

**Abb. 2:**
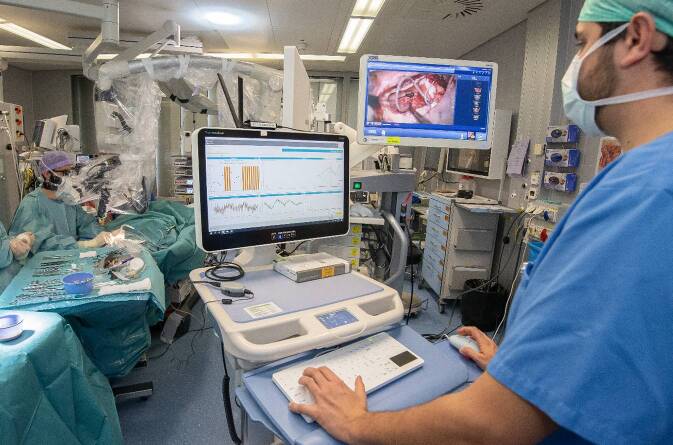
Elektrocochleographie-Messungen während einer Cochleaimplantation. Erläuterung s. Text. (Mit freundl. Genehmigung © Gianni Pauciello, alle Rechte vorbehalten)

Bei der intraoperativen Durchführung der ECochG (Abb. 2) ist entscheidend, dass der Aufbau korrekt und sorgfältig erfolgt, um aussagekräftige Daten über die Innenohrfunktion zu erhalten [3]. Die intracochleäre ECochG erfolgt über das übliche Implantate-System. Bei der extracochleären Messung wird meist eine handelsübliche Elektrode eines Neuromonitoringsystems verwendet. Diese Elektrode wird entweder an das runde Fenster für Messungen vor der Insertion [12] oder auf das Promontorium und damit in unmittelbarer Nähe der basalen Windung der Cochlea für Messungen während der Insertion platziert [13]. Ein Nachteil der extracochleären Messungen ist die teilweise herausfordernde Platzierung der Messelektrode. Die richtige Platzierung kann, meist abhängig von den Platzverhältnissen im Bereich der posterioren Tympanotomie, eine gewisse Zeit in Anspruch nehmen. Bei beiden Messmethoden ist die benötigte Hardware i. d. R. dieselbe, die auch für Impedanzmessungen oder elektrisch evozierte Summenaktionspotenziale genutzt wird. Bei der Software handelt es sich, je nach Hersteller, um eine Forschungssoftware. Die Signale werden normalerweise von Experten aufgezeichnet und sofort interpretiert, was verschiedene Herausforderungen mit sich bringen kann.

Während des Eingriffs sollte darauf geachtet werden, dass die Verbindungsspule des Implantats mit einem ausreichend langen Kabel versehen ist. Dieses sollte durch eine sterile Hülle geschützt werden. Nachdem das Implantatgehäuse in der Periosttasche platziert ist, wird die Spule steril auf die Haut des Patienten aufgesetzt, und der akustische Stimulus wird über einen sterilen Ohrstöpsel abgegeben. Dabei ist darauf zu achten, dass der Schallschlauch keine Knickstellen aufweist und der Ohrstöpsel korrekt im Gehörgang platziert ist, um eine ungeminderte Stimulusintensität sicherzustellen [13]. Je nach Messaufbau kann auch zusätzlich ein Mikrophon im Gehörgang platziert werden, um die tatsächlich im Gehörgang vorhandene Intensität aufzuzeichnen [14].

Vor Beginn der Messung ist zu prüfen, dass die Messelektrode eine niedrige Impedanz aufweist. Zusätzlich sollte darauf geachtet werden, dass das Operationsfeld trocken ist, um Störungen der Messung durch nachfließendes Blut zu vermeiden. Schuerch et al. haben Faktoren aufgelistet, die während einer Operation berücksichtigt werden sollten, um eine uneingeschränkte und kontinuierliche ECochG-Messung zu ermöglichen [3, 15].

Für postoperative ECochG-Messungen gestaltet sich der Ablauf unkomplizierter. Hierbei werden dieselben Komponenten verwendet, allerdings ohne die Notwendigkeit der Sterilität. Vor der Messung sollte überprüft werden, ob der Gehörgang frei ist. Zudem sollten die Stimulusintensitäten nicht im Unbehaglichkeitsbereich liegen. Eine schrittweise Intensitätssteigerung und das Nachfragen beim Patienten sind dabei sinnvoll. Zu beachten ist, dass die Hör- und ECochG-Schwelle zwar korrelieren, aber nicht deckungsgleich sind. Dies lässt sich u. a. mit den unterschiedlichen Eigenschaften der Stimuli erklären [16].

## Ort der Messungen

In diesem Abschnitt wird näher auf die Unterscheidung zwischen extra- und intracochleärer ECochG-Messungen eingegangen. Mit beiden Messmethoden sind, abhängig von der Patientenpopulation und der verwendeten Messmethode, in 80 % der Fälle oder mehr ECochG-Antworten nachweisbar [3, 17].

Intracochleäre Messungen weisen durchschnittlich 14 dB höhere Amplituden auf als extracochleäre

Ein wesentlicher Unterschied zwischen den Methoden ist, dass intracochleäre Messungen Amplituden aufweisen, die durchschnittlich 14 dB höher sind als die von extracochleären Messungen, da sie näher an den Signalgeneratoren liegen [18]. Dabei bilden intracochleäre Antworten hauptsächlich Signale ab, welche von Signalgeneratoren in unmittelbarer Nähe der Aufnahmeelektrode stammen. Dies ermöglicht eine detaillierte Betrachtung des Zustands der Cochlea an einem spezifischen Ort. Im Gegensatz dazu erfassen extracochleäre Messungen Signale von Signalgeneratoren, die aus größeren Abschnitten der basalen Cochlea kommen. Aus diesem Grund bieten diese Antworten ein umfassenderes Bild des Zustands der Cochlea im basalen Bereich [19].

Im Folgenden geht es zuerst um extracochleäre Messungen, die vor oder während der Insertion der CI-Elektrode durchgeführt wurden. Anschließend werden die Ergebnisse von Studien zusammengefasst, die sich mit intracochleären Messungen während der Insertion und postoperativ beschäftigt haben. Es ist anzumerken, dass sich die große Mehrheit der Studien auf die CM beziehen; auch in den folgenden Erläuterungen wird ausschließlich auf diese Signalkomponente eingegangen.

### Extracochleäre Messungen

#### Vor der Implantation

Die extracochleäre ECochG wird typischerweise im OP vor, während oder unmittelbar nach der Elektrodeninsertion durchgeführt. Im Vergleich zu intracochleären ECochG-Antworten sind postoperative Messungen von extracochleären Antworten oft schwieriger oder nicht machbar, da dies das Platzieren einer Elektrode auf dem Promontorium durch das Trommelfell hindurch erfordert.

Extracochleäre Antworten vor der Elektrodeninsertion sind besonders wertvoll, um eine Einschätzung des cochleären Zustandes *vor* der Implantation zu ermöglichen [12]. Um die Restfunktion quantifizieren zu können, wird die „total response“ berechnet. Hierbei werden die elektrocochleographischen Antworten über einen breiten Frequenzbereich von 250–4000 Hz zusammengefasst. Die Amplitude dieser „total response“ wird dann mit dem Verständnis von Einsilbern 3–6 Monate nach der Operation korreliert. Studien haben gezeigt, dass etwa 50 % der Varianz im postoperativen Einsilberverständnis durch die Amplitude der ECochG-Antwort erklärt werden können [12, 20]. Wenn neben der ECochG-Amplitude auch der Insertionswinkel des Implantats in die Analyse einbezogen wird, lässt sich sogar bis zu 72 % der Varianz erklären [21].

#### Während der Implantation

Extracochleäre Aufnahmen während der Elektrodeninsertion zeigen oft stabile Antworten bezüglich Amplitude und Phase, dies steht im Kontrast zu den intracochleären Messungen, welche variablere Antworten aufweisen können. Studien, in denen extra- und intracochleäre Messungen simultan durchgeführt wurden, verdeutlichen, dass Signalveränderungen in intracochleären Messungen nicht zwangsläufig mit Veränderungen in den extracochleären Antworten einhergehen [18]. Ein deutlicher Rückgang der Amplitude in den extracochleären Antworten während der Insertion wird oft mit einem ausgeprägten intracochleären Trauma und einem plötzlichen Verlust des Resthörvermögens in Verbindung gebracht [13, 22]. Allerdings lässt sich aus stabilen extracochleären Antworten nicht grundsätzlich auf den Erhalt des Resthörvermögens schließen.

Interessant ist, dass wiederholte extracochleäre Messungen während der Insertion zeigen, dass eine etwaige Verringerung der Antwortamplitude meist gegen Ende der Elektrodeninsertion auftritt [23]. Dies steht im Einklang mit den Ergebnissen aus intracochleären Messungen [9]. Diese Beobachtungen sind besonders relevant, da sie Hinweise darauf geben können, in welcher Phase der Implantation das cochleäre Trauma am wahrscheinlichsten auftritt, und sie liefern wichtige Informationen für die Entwicklung schonenderer Insertionstechniken sowie die Überwachung und den möglichen Erhalt des Resthörvermögens.

### Intracochleäre Messungen

Intracochleäre Messungen nutzen das CI selbst als Messelektrode, um die elektrocochleographischen Signale aufzunehmen. Diese Art der Messung kann folglich erst beginnen, nachdem die Elektrode in die Cochlea eingeführt wurde. Ein Vorteil ist, dass postoperative Messungen ohne großen Aufwand möglich sind.

#### Während der Insertion

Intracochleäre ECochG-Messungen während der Insertion werden meistens mit der am weitesten apikal gelegenen Elektrode durchgeführt. Die erste Studie, in der solche Messungen an CI-Patienten durchgeführt wurden, fand 2014 statt [24]. Es wurde zum einen beobachtet, dass mit zunehmender Insertionstiefe die Amplitude der ECochG-Antwort im Durchschnitt zunimmt, aber zum anderen auch, dass das Signal während der Insertion häufig starke Fluktuationen in Bezug auf Amplitude und Phase zeigt. Diese Ergebnisse wurden in zahlreichen weiteren Arbeiten verschiedenster Forschungsgruppen bestätigt [25–27]. Viele der während der Insertion beobachteten Signalveränderungen sind wahrscheinlich auf die Bewegung der Aufnahmeelektrode in der Cochlea zurückzuführen, nicht auf Veränderungen der cochleären Restfunktion selbst [18]. Andererseits zeigen verschiedene Arbeiten, dass ein rascher Amplitudenabfall während der Implantation oft mit einem Verlust der akustischen Restfunktion assoziiert ist [27, 28]. Diese Beobachtung wurde auch in 2 systematischen Übersichtsarbeiten bestätigt [17, 29]. In jüngster Zeit wurde auch untersucht, ob die Verwendung von ECochG während der Elektrodeninsertion zu einem besseren Erhalt der Resthörigkeit führen kann. Während die Ergebnisse von Bester et al. [30] darauf hinweisen, dass ein besseres Erhalten des Resthörvermögens möglich ist, indem man auf Echtzeit-ECochG-Feedback reagiert und ggf. interveniert (z. B. Zurückziehen der Elektrode, Stopp der Insertion), konnten Harris et al. [31] keinen solchen Effekt nachweisen.

Die Interpretation intracochleärer ECochG-Messungen bleibt eine Herausforderung

Die Interpretation intracochleärer ECochG-Messungen bleibt aber eine Herausforderung. Während viele der detektierten Veränderungen durch die Bewegung der Elektrode und die Verteilung der noch intakten cochleären und neuralen Strukturen erklärbar sind, repräsentieren einige der Signalveränderungen auch tatsächliche Änderungen in der cochleären Mechanik oder ein akutes cochleäres Trauma. Es ist oft schwierig, die Ursache der Veränderungen im Einzelfall zu bestimmen.

#### Nach der Insertion

**Abb. 3:**
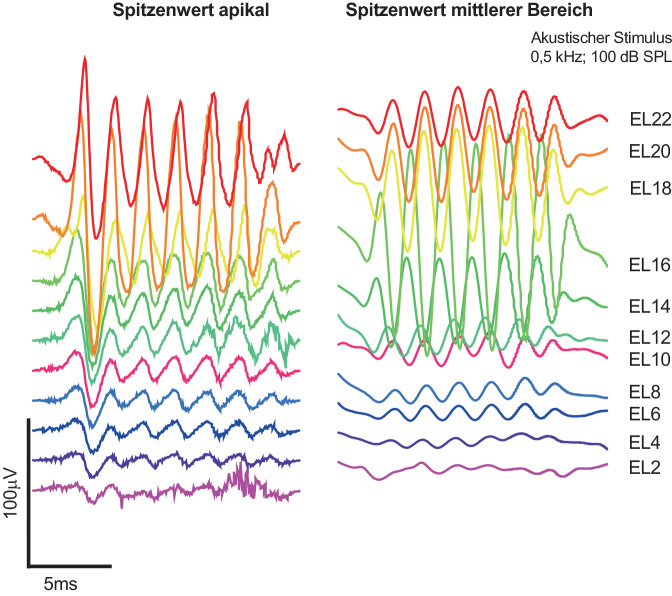
Beispiel für 2 verschiedene Antwortmuster von postoperativen elektrocochleographischen Messungen. Erläuterung s. Text. CM-Antworten an verschiedenen Lokalisationen entlang der Cochleaimplantatelektrode (Elektrode 22, EL 22: apikal; Elektrode 2, EL 2: basal). *Links* Antwortmuster mit der größten Antwort im apikalen Bereich der Elektrode 20, *rechts* maximale Antwort der Elektrode 16 lokalisiert. (Aus [33] Mit freundl. Genehmigung © Wolters Kluwer Health, Inc. Alle Rechte vorbehalten)

In der postoperativen Phase bieten intracochleäre ECochG-Messungen den Vorteil stabiler Messbedingungen, da die CI-Elektrode nicht mehr bewegt wird. Studienergebnisse weisen darauf hin, dass ein Rückgang der ECochG-Amplituden v. a. in der frühen postoperativen Phase auftritt und mit einer Abnahme der Innenohrfunktion korreliert [32]. Eine kürzlich veröffentlichte Studie zielte darauf ab, unmittelbar nach der Einlage auftretende Muster zu identifizieren, die mit einer schlechteren Erhaltung des Resthörvermögens oder einer erhöhten elektrischen Impedanz, d. h. vermehrter intracochleärer Fibrose, zusammenhängen könnten [33]. Dabei wurde festgestellt, dass eine Verschiebung der maximalen ECochG-Amplitude von apikal nach basal oft mit einer schlechteren Resthörerhaltung und einer wahrscheinlich stärkeren intracochleären Fibrose, d. h. erhöhten Impedanzen, einhergeht (Abb. 3). Eine mögliche Erklärung für diese Verschiebung könnte eine Fixierung der Basilarmembran sein, die durch Kontakt zwischen der Elektrode und der Basilarmembran entsteht. Des Weiteren wurde in postoperativen intracochleären Untersuchungen die tonotopische Verteilung der Antworten auf akustische Stimuli mit verschiedenen Frequenzen untersucht [16]. Auf diesen Punkt wird im Weiteren noch etwas genauer eingegangen. In Abb. 3 sind CM-Antworten an verschiedenen Lokalisationen entlang der CI-Elektrode (Elektrode 22, EL 22: apikal; Elektrode 2, EL 2: basal) abgebildet. Auf der linken Seite ist ein Antwortmuster mit der größten Antwort im apikalen Bereich der Elektrode abgebildet (EL 20), auf der rechten Seite ist die maximale Antwort im mittleren Bereich der Elektrode lokalisiert (EL 16).

## Automatisierte Auswertung

Die ECochG-Signalanalyse wird üblicherweise von Experten durchgeführt, was mehrere Probleme mit sich bringt. Für eine korrekte Einschätzung ist umfangreiche Erfahrung nötig. Nicht jedes Hörimplantate-Zentrum verfügt über eine solche Expertise. Darüber hinaus beurteilt jeder Experte die Signalableitungen gemäß seinen eigenen Erfahrungen und Einschätzungen, was eine mangelnde Reproduzierbarkeit mit sich bringt. Dies ist v. a. entscheidend, wenn der Signal-Rausch-Abstand abnimmt oder wenn longitudinale Daten verglichen werden [16]. In der Literatur muss zudem von einem Selektionsbias ausgegangen werden. Dies bedeutet, dass häufig Daten von Patienten mit deutlichen Antwortmustern präsentiert werden, während Patienten mit ungünstigem Signal-Rausch-Verhältnis oder unklaren Antwortprofilen in den Publikationen fehlen. Soll sich ECochG jedoch als Monitoring-Tool der Innenohrfunktion etablieren, müssen alle möglichen Messdaten in die Analysen einbezogen werden. Ein weiteres Problem betrifft die intraoperativen Ableitungen: Die Analyse von ECochG-Signalen kann zeitaufwendig sein, und diese Zeit ist im operativen Setting oft nicht vorhanden. Entsprechend unklar sind die Rückmeldungen an den Chirurgen, oder sie kommen zu spät.

Als Alternative zur Expertenauswertung wurden in den letzten Jahren verschiedene Methoden untersucht, welche die Auswertung der ECochG-Antworten objektivieren und automatisieren [12, 34]. Die Methoden zeigen im Vergleich zur Experteneinschätzung eine ausgezeichnete Diskriminierungsfähigkeit [35]. Der Hauptvorteil der objektiven Analyse liegt darin, dass die Signale objektiv, reproduzierbar und in Echtzeit dargestellt werden können.

Die automatisierte Analyse von ECochG-Signalen ermöglicht eine untersucherunabhängige Bewertung

Zusammenfassend lässt sich sagen, dass die Signalanalyse komplex ist und häufig von der Expertise des Auswertenden abhängt. Diese Abhängigkeit kann durch den Einsatz objektiver Methoden überwunden werden. Die automatisierte Analyse von ECochG-Signalen ermöglicht eine standardisierte, schnelle, präzise und untersucherunabhängige Bewertung. Erst dies macht eine systematische Untersuchung von ECochG-Signalen und den Vergleich zwischen verschiedenen Individuen sowie longitudinale Vergleiche möglich. Zudem sind diese Verfahren in der Lage, Musterveränderungen in Echtzeit zu erfassen, was insbesondere im intraoperativen Kontext von entscheidender Bedeutung ist.

## Radiologische Korrelation und Tonotopie

**Abb. 4:**
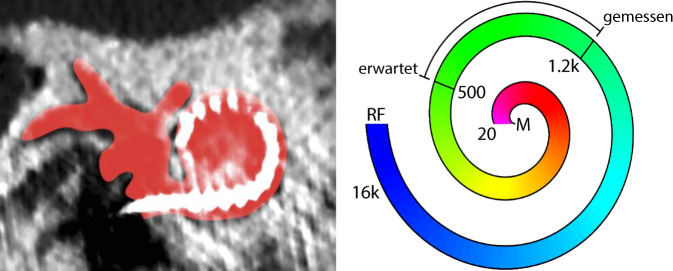
Bestimmung der intracochleären Position der Messelektrode mittels postoperative Computertomographie. Erläuterung s. Text. *M* Modiolus, *RF* rundes Fenster. (Mit freundl. Genehmigung © Raphael Andonie, alle Rechte vorbehalten)

In Studien wurde gezeigt, dass die Länge der Cochlea individuell stark variiert [36]. Darüber hinaus weisen die Elektrodenträger der verschiedenen Hersteller und Modelle unterschiedliche Längen auf. Diese beiden Faktoren beeinflussen die tonotopische Position, an der die Messung erfolgt [37]. Aus Sicht der Autoren ist es essenziell, die exakte intracochleäre Position, an welcher die ECochG-Messung durchgeführt wird, zu spezifizieren. Dies ist die Voraussetzung für ein vertieftes Verständnis der intracochleären Signale. Die Positionierung der Elektrode kann einerseits durch eine postoperative Computertomographie (CT) identifiziert werden, die eine Schätzung der tonotopischen Frequenz der Messelektrode ermöglicht ([16]; Abb. 4). Liegt keine postoperative Bildgebung vor, lässt sich die Position alternativ durch die Analyse der Impedanzwerte abschätzen [38]. Wie in Abb. 4 dargestellt, lässt sich mit der postoperativen CT die intracochleäre Position der Messelektrode bestimmen. Die tonotopische Frequenz dieser Position kann anschließend mithilfe der Greenwood-Funktion berechnet werden. Dies ermöglicht den Vergleich der größten gemessenen Signalamplitude mit der erwarteten intracochleären Messposition.

Das Wissen um die Frequenz, bei der die ECochG-Messungen durchgeführt werden, trägt zur Verbesserung des Verständnisses der beschriebenen ECochG-Muster bei. So wurde in objektiv analysierten ECochG-Daten festgestellt, dass die größte ECochG-Signalamplitude häufig weiter basal liegt als erwartet [16]. Dieses Phänomen könnte durch verschiedene Faktoren bedingt sein: Zum einen ist es möglich, dass durch eine hohe Stimulusintensität auch basale Haarzellen erregt werden [7]. Zum anderen könnte die Implantatelektrode die elektrophysiologischen Eigenschaften der Basilarmembran verändern [33]. Eine weitere Hypothese ist, dass in den basalen Bereichen der Cochlea vermehrt Signalgeneratoren präsent sind, die das Signal beeinflussen [39].

Zusammenfassend ist zu betonen, dass die Kenntnis der exakten tonotopischen Messposition unabdingbar für das Verständnis der intracochleären Vorgänge ist und stets eine entsprechende Berechnung oder Schätzung der Position erfolgen sollte.

## Ausblick

Festgehalten werden kann, dass die ECochG ein vielseitiges diagnostisches Verfahren ist, welches wertvolle Einblicke in cochleäre Antworten und Veränderungen vor, während und nach der Cochleaimplantation bietet. Sowohl die Aufnahme der Signale im OP als auch deren Interpretation können herausfordernd sein. Viele Aspekte, insbesondere der zahlreichen Signalveränderungen während der Elektrodeneinlage, sind zzt. noch kontrovers und können nicht klar zugrunde liegenden Mechanismen zugeordnet werden.

Ein rascher Amplitudenabfall während der Insertion ist mit schlechterem Resthörvermögen assoziiert

Dennoch ist die Assoziation eines raschen Amplitudenabfalls während der Insertion mit schlechterer Erhaltung des Resthörvermögens ein konsistenter Befund, der sowohl in intra- als auch in extracochleären Aufnahmen beobachtet wurde.

Die postoperativen ECochG-Messungen zeigen ein großes Potenzial für die Zukunft, da sie helfen könnten, Vorgänge innerhalb der Cochlea nach der Implantation besser zu verstehen und frühzeitig Veränderungen zu erkennen, die beispielsweise zu verstärkter Fibrose oder erheblichem Verlust der Resthörigkeit führen könnten. Die weitere Erforschung und ein tieferes Verständnis der den ECochG-Veränderungen zugrunde liegenden Mechanismen sind entscheidend. Ebenso ist die Automatisierung und Objektivierung der Signalauswertung ein wichtiger Schritt, der die Anwendbarkeit und Verbreitung der ECochG im Rahmen der Cochleaimplantation weiter fördern könnte. Durch die stetige Verbesserung und Anpassung dieser Methoden könnten Präzision und Zuverlässigkeit der ECochG-Messungen erhöht und somit die Behandlung und Betreuung von CI-Patienten weiter verbessert werden.

Ein weiterer wichtiger Schritt wird die Korrelation elektrocochleographischer Messungen mit anderen Messmethoden sein. Dabei sind elektrophysiologische Messmethoden, die auf elektrischer anstatt akustischer Stimulation beruhen, sowie verschiedene Formen von Impedanzmessungen zu berücksichtigen. Die Korrelation mit anderen Messmethoden könnte in Zukunft eine deutlich detailliertere Einschätzung der noch vorhandenen und stimulierbaren cochleären und neuralen Strukturen erlauben und damit auch eine bessere Prognose bezüglich des CI-Outcomes ermöglichen.

## Fazit für die Praxis

- Die Elektrocochleographie (ECochG) ist ein vielseitiges diagnostisches Verfahren, welches wertvolle Einblicke in die cochleäre Funktion und in Veränderungen dieser Funktion vor, während und nach der Cochleaimplantation bietet.
- Bei der Durchführung der ECochG ist es entscheidend, dass der Aufbau korrekt und sorgfältig erfolgt, um aussagekräftige Daten über die Innenohrfunktion zu erhalten.
- Ein rascher Abfall der Amplitude in der ECochG während der Insertion ist mit einer schlechteren Erhaltung des Resthörvermögens assoziiert.
- Die postoperativen ECochG-Messungen könnten in Zukunft dabei helfen, Vorgänge innerhalb der Cochlea nach der Implantation besser zu verstehen.
- Die Automatisierung und Objektivierung der Signalauswertung könnten die Anwendbarkeit und Verbreitung der ECochG im Rahmen der Cochleaimplantation weiter fördern.
